# Pressure Sensors for Measuring the Grip Pressure during Kendo Attacks: Assessment of Laterality and Evidence of the Five Phases of Attack

**DOI:** 10.3390/s23031189

**Published:** 2023-01-20

**Authors:** Kwangyul Jeong, Adin Ming Tan, Takeshi Asai, Kunihide Koda, Franz Konstantin Fuss

**Affiliations:** 1 Chair of Biomechanics, Faculty of Engineering Science, University of Bayreuth, D-95440 Bayreuth, Germany; 2 Smart Equipment Engineering and Wearable Technologies Research Program, Swinburne University of Technology, Melbourne, VIC 3122, Australia; 3 Faculty of Health and Sports Sciences, University of Tsukuba, Tsukuba 305-0821, Japan

**Keywords:** pressure sensor, Kendo, Shinai, grip pressure, attack phases, left-hand pressure, right-hand pressure

## Abstract

In Kendo, there is no consensus as to which hand should produce more pressure when attacking the opponent with the bamboo sword, let alone how to teach the pressure distribution during coaching. There is the theory that a Kendo attack can be divided into five phases, which has not entered the coaching practice, either. The aim of this study was to measure the grip pressure during Kendo attacks, investigate the pressure distribution between the two hands, and find evidence for the existence of the alleged five attack phases. We instrumented a bamboo sword with grip pressure sensors and investigated the grip pressure in 23 participants. In all attack targets and in both hands, the pressure across all attack phases was significantly different. In general, the left-hand pressure was consistently and significantly higher than the right-hand one, across all five attack phases, for the hand, head, and flank attack targets. The surprising exception was the throat target with only two attack phases, the strike phase of which showed a greater pressure in the right hand. Across all participants, the left-hand pressure was greater in 60.22–100% in any phase of the four attack targets, except for the strike phase of the throat target. Through these results, we could verify the effect of the teaching customs in Kendo, as well as provide first-time evidence of the existence of the five attack phases.

## 1. Introduction

**Figure 1:**
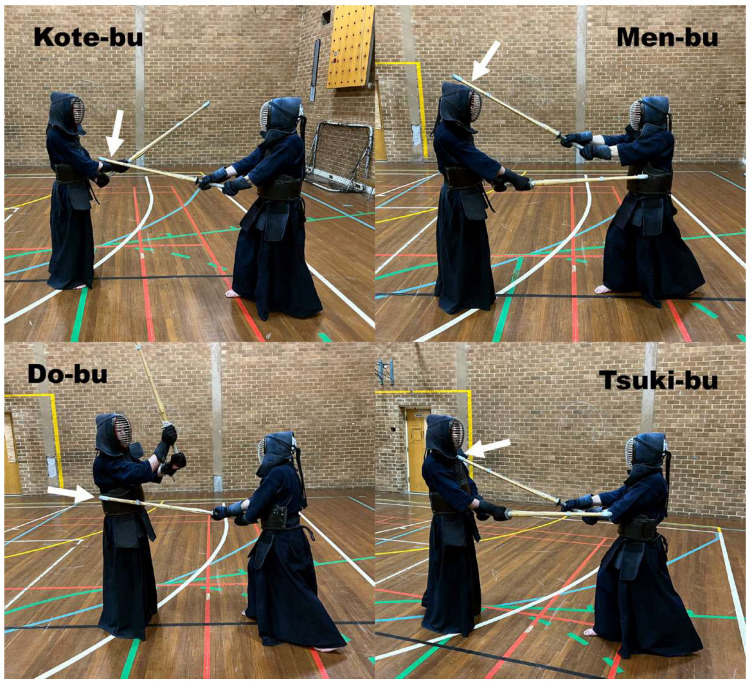
The four scoring targets in Kendo: wrist (Kote-bu), head (Men-bu), flank (Do-bu), and throat (Tsuki-bu-bu).

Kendo, the ‘way of the sword’ is one of the most ancient and traditional Japanese martial arts with a full contact fight using bamboo swords (Shinai), which dates to the swordsmanship of the ancient Samurai. According to the rules of competition, scoring two points is required for winning a Kendo match. Scoring a point in a Kendo competition is defined as an accurate strike or thrust against a point-scoring target of the opponent’s armour resulting in physical contact with the correct part of the Shinai [1]. In addition, the umpire (Shinpan) will also consider a high level of fighting spirit, correct stance and posture, and continuing awareness (Zanshin [1]) as inherent components of scoring. There are four scoring targets in Kendo (Figure 1), which are Kote-bu (wrist), Men-bu (head), Do-bu (flank), and Tsuki-bu-bu (throat). Hitting each target correctly is worth one point. 

The basic stance (Kamae) usually places the left foot behind the right foot, both feet pointing forward, with both hands holding the grip surface of the Shinai (right hand on top of left hand). This stance was reported to contravene the fundamental rule of sports, namely the diagonal-sequence arm-leg movements or “when the right foot moves forward then the left arm moves forward” [2]. However, in Kendo, the attack from the standard Kamae (Chudan no Kamae) leads to the forward movements of both the right foot and arm [2]. 

To the best of the authors’ knowledge, there is no English literature in Kendo explicitly teaching how the grip pressure should be distributed on the Shinai. Techniques are usually taught verbally, and different instructors have their own theories. Therefore, to generalise the common Kendo teaching and coaching practice, and understanding for this research, a questionnaire on grip pressure in Kendo teaching [3] was carried out with Kendo instructors (Sensei) of the 6th Dan and above. All the Sensei(s) had a practicing experience of a minimum of 27 years in Japan, Korea, or Australia. The results of the survey [3] have shown that 86% of them believed that it is essential to emphasise the importance of grip pressure when teaching their students, i.e., how tight the grip should be when holding the Shinai). A total of 71% of them believed that the left-hand grip pressure is generally higher than the right-hand one, and there is a change in grip pressure distribution between the left and right hand from Kamae to the impact motion aiming at the four principal targets (Kote-bu, Men-bu, Do-bu, and Tsuki-bu). The majority (57%) believes that the grip pressure of the left hand is high during Kamae and the right-hand grip pressure increases close to the impact. The grip pressure is believed to peak exactly at the impact [3].

Digital technology in sport [4] has revolutionised performance analysis and coaching by using smart sporting goods equipped with sensors [5]. There are several studies available that utilise Smart Shinais. 

Takata et al. [6] instrumented the Shinai with two inertial measurement units (IMUs; and the Kendoka with further IMUs) for activity analysis and classification of scoring targets. Yotani et al. [7] instrumented a Shinai with strain gauges and compared the deflection of Shinai to the muscle activation patterns.

Hayashi [8] installed a Shinai connected to a PCD-300A (Kyowa Electronic Instruments Co., Ltd., Tokyo, Japan) interface to measure the grip pressure of the participant’s hands during Kendo attack motions. He reported the difference in grip pressure between the Kihon-uchi (large practice cut) and Jissen-uchi (competition-style cut, fast small cut). The results showed that the grip pressure of Jissen-uchi was 2.1 times stronger than Kihon-uchi. The right to left hand grip pressure ratios were 2.4 for Kihon-uchi and 1.5 for Jissen-uchi [8].

Another study carried out by Watanabe et al. [9] utilised the same technology as Hayashi [8] did. They reported that in Kihon-uchi cuts, the right hand applied 1.5–2.0 times more grip force compared to the left. For Jissen-uchi cuts, the right-hand pressure was 0.9–1.5 times stronger than the left hand one [9].

Shibata et. al. [10] carried out a study of the Tenouchi motion (Japanese term used in Kendo teaching, addressing the use of wrist and hand grip to manoeuver the Shinai effectively) in comparison to motions of hitting a ball. Utilising the same technology as Hayashi [8] did, it was reported that there was no technical difference between experienced Kendo practitioners and beginners in Tenouchi when hitting a ball with the Shinai. 

In a previous study [11], a smart Kendo sword was developed with in-house-built pressure sensors on the Shinai grip surface for individually assessing the pressure of the left and right hands. The study carried out was to investigate the grip pressure pattern during Kamae (basic stance) and Kote-bu cut (striking at the hand target) by a professional Kendo athlete. It was reported that the grip pressure of the left hand was significantly higher compared to the one of the right hand in the Kamae stance. However, at the impact point, the grip pressure of both hands increased considerably and the grip pressure difference between left and right hand reduced significantly at this stage [11]. This study provides evidence that the grip pressure changes during the different stages of attack in Kendo.

The Kendo attack action (before, during, and after striking with the Shinai) is divided into five different attack phases [12,13,14,15]:

(1) Kamae (構える, “to hold”), referring to the basic stance and the way of holding the bamboo sword (Shinai) before an attack and between consecutive attacks;
(2) Seme (攻める, “to attack”), referring to the initiation of an attack by applying mental or physical pressure to the opponent as a result of the imminent attack;
(3) Toraeru (捉える, “to catch”), referring to ‘catch an opportunity’ or take advantage of the attack opportunity;
(4) Datotsu (打突する, “to strike”), referring to eventually executing the attack through the physical impact between the attacker’s Shinai and the target on the opponent’s bogu (armour); and
(5) Zanshin (残心を示す, “ambition”), referring to the follow through after impact, thereby exhibiting a good level of fighting spirit and being prepared for the opponent’s counterattack.

To the best of the authors’ knowledge, there is no scientific evidence available in the literature proving that these five attack phases really exist.

The aim of this study is therefore twofold: (1) to measure the magnitude and the distribution of the grip pressure of left and right hands during the basic stances and different Jissen-uchi competition-style cuts at the four scoring targets, and (2) to identify the five attack phases from the sequence of the changes in pressure. Due to the underlying theory of Kendo, this study is inherently *hypothesis-driven*. The originality and contribution to the literature of this study are (1) a detailed analysis of the grip pressure distribution when hitting the four scoring targets, and (2) first-time scientific evidence that the alleged five attack phases really exist.

The associated hypotheses to be tested are

**Hypothesis 1** **(H1).** *The grip pressure varies significantly during the attack and, as such, different attack phases could be identified and distinguished directly from the changing grip pressure. The rationale for this hypothesis is that the motion of the Shinai can also be divided into different stages, too, namely, the Kamae, Seme, Toraeru, Datotsu, and Zanshin, at least for the three attacks carried out with a striking motion (Kote-bu, Men-bu, Do-bu)*.

**Hypothesis 2** **(H2).** *The grip pressure of the left hand is significantly greater than the one of the right hand. The rationale for this hypothesis is that most Kendo instructors believed that the Kendokas should focus on the left hand rather than on the right hand* [3].

**Hypothesis 3** **(H3).** *The magnitude of the grip pressure distribution varies across the four scoring targets. The rationale of this hypothesis is based on the type of the Shinai’s motion: thrust (linear acceleration) for Tsuki-bu target; strike (angular acceleration) for Do-bu, and thrust followed by strike for Kote-bu and Men-bu. Therefore, it is expected that the grip pressure is at its maximum when attacking the Kote-bu and Men-bu targets*.

**Hypothesis 4** **(H4).** *Even if it is hypothesised that the grip pressure of the left hand is significantly higher than the one of the right hand (Hypothesis 2), this may not necessarily be true for a single attack phase. It could very well be that in a single specific phase the right-hand pressure is greater than the left hand one. The same could apply to the grip pressure distribution across the different players, such that some have a greater left-hand pressure and others a greater right-hand pressure. It is expected and therefore hypothesised, though, that in most attack phases, across the four scoring targets and across all the players investigated, the left hand is the dominant one. It is further expected that most players prefer to produce a greater pressure with their left hand. While Hypothesis 2 refers to averaged data, Hypothesis 4 applies to the specific variation of the grip pressure*.

In summary,

- Hypothesis 1 tests the existence of the five different attack phases within the four different scoring targets and their consistency across the Kendokas, through detecting significant variations of the grip pressure magnitude across the attack phases;
- Hypothesis 2 tests the significance of the left-hand pressure being greater than the right-hand pressure;
- Hypothesis 3 tests the variation of attack-phase pressure across the four scoring targets; and
- Hypothesis 4 tests the laterality across the attack phases, scoring targets, and players in terms of the majority of increased left-hand pressure events.

## 2. Materials and Methods

### 2.1. Smart Shinai Development

**Figure 2:**
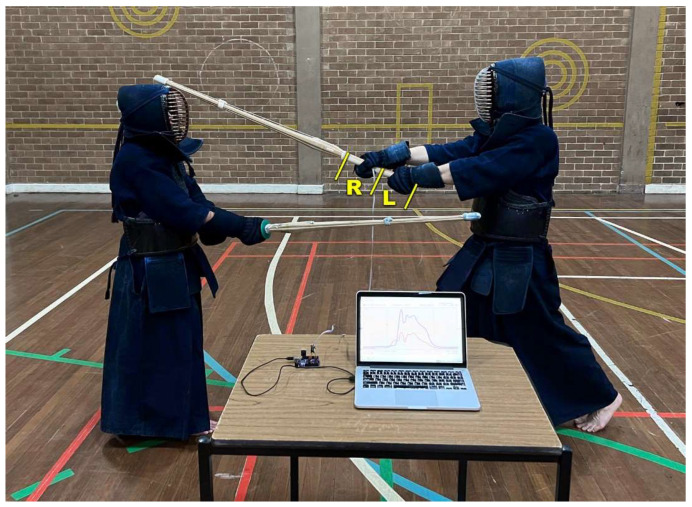
Experimental setup for testing the grip force with Smart Shinai (Men-bu, head target); L = pressure sensor of left hand, R = pressure sensor of right hand.

**Figure 3:**
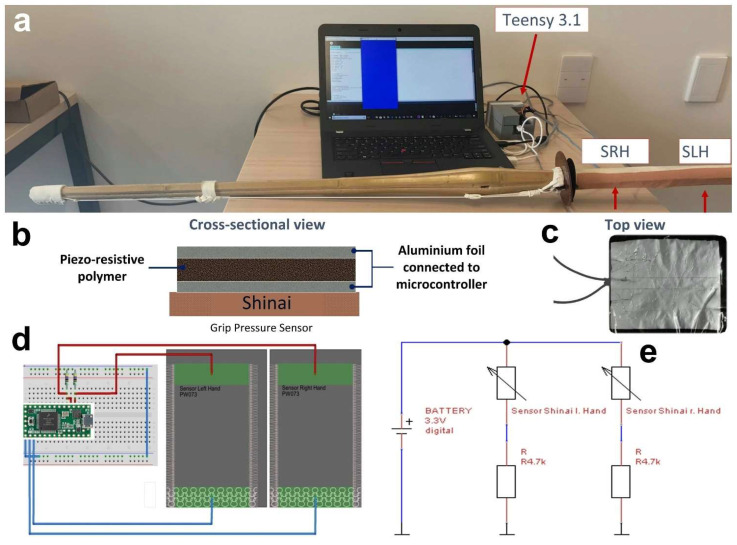
Development of the grip pressure sensor; (**a**) sensor system components; (**b**) schematic cross section of the sensor; (**c**) polymer pressure sensor with top electrode; (**d**,**e**) circuit diagrams with voltage dividers.

In the standard Kendo stance (Chudan no Kamae), the practitioner uses both hands to hold the Shinai (Figure 2) at the Tsukagawa (grip/handle) with the right hand closer to the Tsuba (hand guard) and the left hand closer to the butt of the handle (Tsukagashira). To measure the grip pressure of both hands, a standard Shinai was equipped with two pressure sensors (Figure 3), one for each hand, like the one developed by Jeong et al [11]. The two 135 mm × 105 mm sensors were made of a piezo-resistive polymer sensor material sandwiched between alumina foil electrodes. The sensor material selected for pressure measurements was an electrostatic carbon-infused polymer (Velostat, 3M, St. Paul, MN, USA) with piezo-resistive properties, applied in previous research projects [16,17,18] because of its accuracy and cost-effectivity. The polymer matrix of this material is made of polyolefin [16,19], and the electrical resistivity of this material in the unloaded state is approximately 23 kΩm [18]. Each sensor was connected to a voltage divider (Figure 3) with a reference resistor of 4.7 kΩ. The drop voltage *V_ref_* was measured across the reference resistor by a Teensy 3.1 (LLC, Sherwood, OR, USA) and recorded at a sampling frequency of 50 Hz. The Teensy was powered by, and connected to, a laptop. The cables connecting the sensors to the electronics unit were placed inside the sleeve of the Kendo coat (keiko-gi) and therefore did not obstruct any movement.

### 2.2. Data Processing

The raw ASCII data were obtained from the Teensy with a 10-bit ADC, and their values ranged from 0 to 1023. The analogue-to-digital units (ADU) data were converted to voltage drop across the reference resistor (*V_ref_*).

(1) $$V_{ref}=V_{in}\times \frac{ADU}{1023}$$

where *V_in_* is the input voltage (3.3 V) and *V_ref_* is the voltage drop across the reference resistor. The resistance *R_sensor_* of each sensor was calculated from the voltage divider equation:

(2) $$R_{sensor}=\frac{V_{in}\cdot \;R_{ref}-V_{ref}\cdot R_{ref}}{V_{ref}}$$

For the piezo-resistive sensors used in this study, the pressure, *p*, is a power function of the conductance [16] *G*, where *G* = 1/*R*. As the calibration function of the selected piezo-resistive material has a power exponent of slightly greater than unity [16], partial loading (which happens when gripping the sensor) does not cause a substantial error with respect to the even pressure distribution [16].

The two sensors were calibrated with an Instron material testing machine (model number 5967, MA, USA) and simultaneously recorded the drop voltage across the reference resistor (4.7 kΩ). The force obtained from the material testing machine and the drop voltage obtained from the reference resistor were converted to pressure and conductance, respectively, and subsequently fitted with a power function which was used for establishing the calibration curve, i.e., for calculating the pressure from conductance.

After mounting the sensors on the Shinai’s handle, the calibration curves were validated with the worst case of partial loading, by placing masses of 1, 5, and 10 kg on each sensor section of the handle, placed on a Kistler force plate (type 9260AA6, Kistler Instruments, Winterthur, Switzerland). The force measured by the force plate was doubled (top reaction force from the mass and bottom reaction force from the force plate) and divided by the sensor area to obtain the average pressure across the force plate. The pressure calculated from the calibration curve was slightly less than the pressure calculated from the force plate data, which was expected, as the power exponent of the calibration curves was slightly higher than unity.

### 2.3. Association of the Attack Phases with the Grip Pressure

The Kamae (basic stance) phase was recognised from the continuous pressure pattern with low magnitude. 

The Datotsu phase was identified from initially placing a third pressure sensor on the Kote glove, by performing a standard striking motion for hitting the Kote-bu. We recorded the data of all 3 sensors simultaneously. The 3rd sensor on the glove recorded the impact pressure of the Shinai’s tip only. The impact spike of the third sensor allowed for identifying the changes in grip pressure of the two hands during impact. As we were able to locate this impact spike on the pressure sensor signal, recorded from the grip sensors, too, there was no need for the 3rd sensor anymore.

The Zanshin phase was located between the impact spike and the subsequent Kamae phase.

The identification of the Seme phases was expected from the increasing pressure to be verified from the experimental data. How to identify the Toraeru phase was determined statistically from the pressure signals.

### 2.4. Experimental Procedure

Twenty-three experienced male Kendokas (holding 2nd Dan to 8th Dan) participated in this study. Their age was 22 yr (median) with an IQR of 3 yr (19–52 yr, range 33 yr); body mass 75.1 ± 10.3 kg (58-93, range 35 kg); body height 174.9 ± 5.4 cm (165-187, range 22 cm); BMI 24.5 ± 2.9 kg/m^2^ (19.6–30.7, range 11.1 kg/m^2^); Dan rank 3 (median) with an IQR of 1 (2–8, range 6); training experience 14 yr (median) with an IQR of 7 yr (4–40 yr, range 36 yr). The participants had no known musculoskeletal pathologies and showed no sign of pain that could affect their competitive performance.

The participants were asked to use the Smart Shinai to perform four different attacking motions on the four targets (Kote-bu, Men-bu, Do-bu, and Tsuki-bu). They were requested to repeat the same attack 10 times on each of the targets with an approximate 5 s pause in between each strike. The grip pressure was recorded for 10 measurements per target by each Kendoka (Figure 2). All 10 measurements per Kendoka were processed without any exclusions. The participants were not briefed regarding the commonly accepted opinion that the left-hand pressure should be higher than the right-hand pressure, and rather were instructed to carry out the attack in the way they used to do during the competitions. Equally, the participants were neither informed of the theory of the 5 attack phases, nor were the 5 attack phases mentioned at any point during the experiments. Thus, although the attacks represented a staged scenario, the technique the Kendokas used reflects both teaching practice and experience they gathered from competitions, a technique that is therefore shaped by theory and practice. This study was granted ethics approval by the Swinburne University Human Ethics Committee (approval no. 2016/296) and adhered to the Declaration of Helsinki. An informed consent form was filled in by all the participants before the start of the experiment. 

### 2.5. Data Processing and Statistics

From the pressure data obtained from the experiments, we identified the different attack phases, and the maximum or minimum pressure datum of each phase was recorded for statistical analysis.

For testing Hypotheses 1 and 3, the following method was applied.

As the pressure data were skewed towards higher pressures, non-parametric tests were used for data comparison. Right- and left-hand pressure data were compared with the Wilcoxon signed-rank test for each attack technique, as these data were recorded simultaneously. The grip pressures of the different phases of attack were compared with the Kruskal–Wallis test, and the significance of the individual differences of the five phases of attack as well as of the four different scoring targets was assessed with the Conover and Dunn post-hoc tests, both of them adjusted by the Holm FWER (familywise error rates) and Benjamini–Hochberg FDR (false discovery rate) methods. The effect size was calculated in terms of the Rank-Biserial Correlation [20], *r*, from the U-value *r* = |1 − 2*U* / (*n*_1_ *n*_2_)|, where *n*_1_ and *n*_2_ denote the amount of data compared by the Mann–Whitney test. Note that the effect size *r* ranges from zero to unity. The effect size *r* was interpreted according to the recommendations of McGrath and Meyer [21] (very small: 0 ≤ *r* < 0.1, small: 0.1 ≤ *r* < 0.24, medium: 0.24 ≤ *r* < 0.37, large: 0.37 ≤ *r* < 1).

For testing Hypothesis 2, the pressures of the left and the right hands were compared for all attack phases and techniques with the Mann–Whitney test. The effect sizes were calculated in the same way as for Hypothesis 1. 

For testing Hypothesis 4, three different methods were used (i.e., pressure difference between left and right hand; individual phases versus combined phase; and player-specific data):

(a) The pressure differential Δ*p*_L–R_ between left and right was calculated, and the number of instances with the left-hand pressure greater than the right-hand pressure across all phases per attack technique of 10 attacks per technique and participant were determined as a percentage of the total number of phases (n = 230; 10 attacks and 23 participants). The weighted number of instances was calculated from the sum of positive Δ*p*_L–R_ divided by the sum of |Δ*p*_L–R_| times 100 (as a percentage). The weighted number served to determine whether |Δ*p*_L–R_| is greater if Δ*p*_L–R_ is positive (i.e., left-hand pressure greater than right-hand pressure).

(b) It was explored in how many phases per attack technique the pressure differential was positive or negative. This resulted in the following main combinations:

- all 5 phases: *p*_R_ > *p*_L_
- 4 phases: *p*_R_ > *p*_L_; 1 phase *p*_L_ > *p*_R_
- 3 phases: *p*_R_ > *p*_L_; 2 phases *p*_L_ > *p*_R_
- 3 phases: *p*_L_ > *p*_R_; 2 phases *p*_R_ > *p*_L_
- 4 phases: *p*_L_ > *p*_R_; 1 phase *p*_R_ > *p*_L_
- all 5 phases: *p*_L_ > *p*_R_

There are further possible combinations that involve equal pressure of the right and left hands in at least one phase. It seems to be highly unlikely, however, that equal pressure distribution will occur as the pressure values were rounded to 0.1 kPa. The number of phases per combination was expressed as a percentage (n = 230). 

(c) The players’ pressure distribution was explored by calculating the percentage of players that exhibited a greater left-hand pressure compared to the right-hand pressure. The percentages were grouped in 4 quarters (left-hand pressure greater in 0–25% of all players for a specific attack technique, 26–50%, 51–75%, 76–100% of all phases). 

## 3. Results

**Figure 4:**
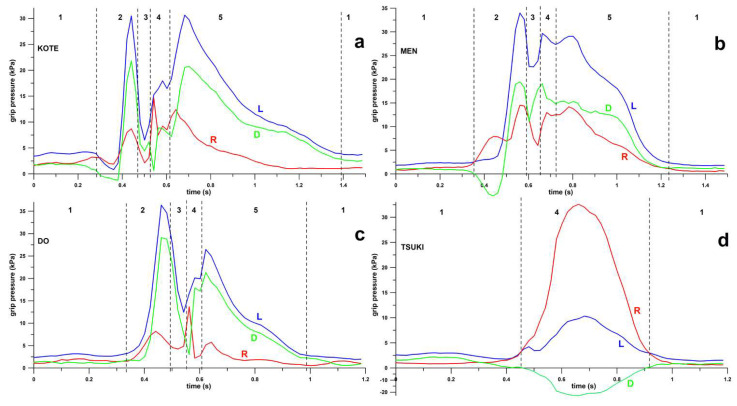
Example of grip pressure profile (pressure vs. time) of a participant hitting four different targets (**a**) Kote-bu, (**b**) Men-bu, (**c**) Do-bu, and (**d**) Tsuki-bu measured by the Smart Shinai; L = left hand, R = right hand, D = pressure differential (L–R); 1–5: attack phases; 1 = Kamae, 2 = Seme, 3 = Toraeru, 4 = Datotsu, 5 = Zanshin.

**Figure 5:**
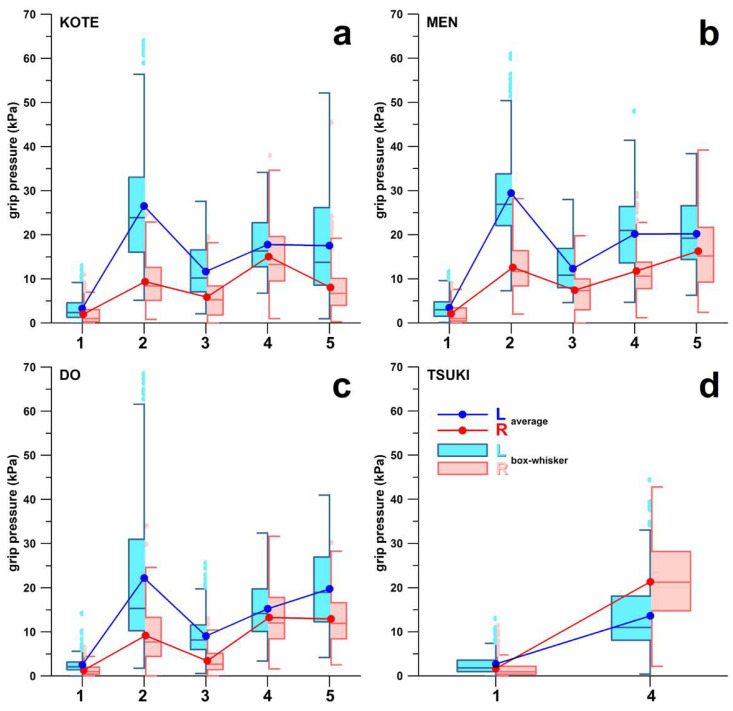
Box and whisker plot of the grip pressure profiles including the mean values across five attack phases (1-5) of 23 participants hitting four different targets (**a**) Kote-bu, (**b**) Men-bu, (**c**) Do-bu, and (**d**) Tsuki-bu measured by the Smart Shinai; L = left hand, R = right hand; 1 = Kamae, 2 = Seme, 3 = Toraeru, 4 = Datotsu, 5 = Zanshin.

The varying pressure across the attack phases and four scoring targets are shown in Figure 4. Box and whisker plots of the grip pressure of the different phases are shown in Figure 5.

### 3.1. Hypothesis 1

In the Kote-bu, Men-bu, and Do-bu targets, we found all five attack phases, whereas in the Tsuki-bu target, only two phases were present (Kamae and Datotsu). 

In the Kote-bu, Men-bu, and Do-bu target techniques, in both the left and the right hand, the pressure of all five attack phases was significantly different (Kruskal–Wallis *p* < 0.001).

In the Kote-bu target, the post-hoc *p*-values were smaller than 0.04. Datotsu and Zanshin seemed to have similar averages in Figure 5a; however, their mean ranks and medians were significantly different. 

In the Men-bu target, the post-hoc tests revealed that there was no significant difference between Datotsu and Zanshin in the left hand, or between Seme and Datotsu in the right hand. In the right hand, Seme and Datotsu phases were separated by the Toraeru phase, the magnitude of which is significantly different from the preceding and following phases. Although pressures of the Datotsu and Zanshin phases in the left hand did not show any significant difference, they were nevertheless separated by a slight decrease in pressure between the two phases (Figure 5b), such that they could be distinguished clearly.

In the Do-bu target, the post-hoc tests indicated that there was no significant difference between Seme and Zanshin (and also Seme and Datotsu in the Dunn test only) in the left hand, or between Datotsu and Zanshin in the right hand. In the left hand, the Seme and Zanshin or Datotsu phases were separated at least by the low-pressure Toraeru phase; in the right hand, Datotsu and Zanshin phases were still separated by a slight decrease in pressure (Figure 5c).

In contrast to the three previous scoring targets, there were only two attack phases found in the Tsuki-bu target (Figure 5d), expressed as a single Datotsu pressure spike. In both the left and the right hand, the Kamae pressure and the Datotsu pressure were significantly different (Kruskal–Wallis *p* < 0.001).

Hypothesis 1 could therefore be confirmed insofar that the grip pressure across the attack phases was mostly significantly different, and that based on the grip pressure, the different attack phases could be clearly identified and distinguished. 

### 3.2. Hypothesis 2

Regarding Hypothesis 2, for Kote-bu, Men-bu, and Do-bu targets, the pressure exerted by the left hand was consistently and significantly (*p* < 0.002) higher than the one produced by the right hand, and this was across all five attack phases. 

In the Kote-bu and Do-bu targets, *p* < 0.001; and the effect sizes were large (0.375 < r < 0.789) in all attack phases but one (Datotsu, r = 0.226 and 0.190, respectively, small effect size).

In the Men-bu target, *p* < 0.001; and the effect sizes were large (0.411 < r < 0.895) in all attack phases but one (Zanshin, r = 0.283, medium effect size).

In the Tsuki-bu target, the left-hand grip pressure of the Kamae was higher than the one of the right hand (*p* < 0.001; r = 0.348, medium effect size). In contrast to this, unexpectedly and surprisingly, the right-hand grip pressure of the Datotsu phase was higher than the one of the left (*p* < 0.001; r = 0.511, large effect size). 

Hypothesis 2 could therefore be confirmed, namely, that the grip pressure of the left hand was greater than the one of the right hand in all attacks and phases but one, namely, the Datotsu phase of the Tsuki-bu target.

### 3.3. Hypothesis 3

Regarding Hypothesis 3, the pressure of a specific attack phase out of the five phases being different across three scoring targets (Kote-bu, Men-bu, Do-bu), the result was indeed mostly significantly different, with some exceptions.

The pressure of the left hand in the Kamae phase was, for the Do-bu target (median pressure 2.1 kPa), significantly (*p* < 0.003) smaller than for Kote-bu (2.4 kPa) and Men-bu (3 kPa).

The pressure of the right hand in the Kamae phase was not different between Kote-bu, Men-bu, and Do-bu (all medians were at 1 kPa).

The pressure of the left hand in the Seme phase was significantly different (*p* < 0.001) for Kote-bu (23.9 kPa), Men-bu (26.9 kPa), and Do-bu (15.35 kPa).

The pressure of the right hand in the Seme phase was significantly (*p* < 0.001) greater for the Men-bu target (11.7 kPa) compared to Kote-bu (8.4 kPa) and Do-bu (7.7 kPa).

The pressure of the left hand in the Toraeru phase was significantly (*p* < 0.001) smaller for the Do-bu target (8.2 kPa) than for Kote-bu (10.2 kPa) and Men-bu (10.85 kPa).

The pressure of the right hand in the Toraeru phase was significantly different (*p* < 0.001) for Kote-bu (5.25 kPa), Men-bu (7.35 kPa), and Do-bu (2.65 kPa).

The pressure of the left hand in the Datotsu phase was significantly different (*p* < 0.001) for Kote-bu (16.35 kPa), Men-bu (21 kPa), and Do-bu (14.2 kPa).

The pressure of the right hand in the Datotsu phase was significantly different (*p* < 0.001) for Kote-bu (13.25 kPa), Men-bu (10.6 kPa), and Do-bu (12 kPa).

The pressure of the left hand in the Zanshin phase was significantly (*p* < 0.001) smaller for the Kote-bu target (13.8 kPa) than for Men-bu (19.2 kPa) and Do-bu (19 kPa).

The pressure of the right hand in the Zanshin phase was significantly different (*p* < 0.001) for Kote-bu (6.7 kPa), Men-bu (15.2 kPa), and Do-bu (11.9 kPa).

Out of 30 possible significantly different pairs, only 7 were not significantly different (23.3%). Hypothesis 3 was therefore confirmed in more than three quarters of all cases.

### 3.4. Hypothesis 4

Regarding Hypothesis 4, in 60.22%–100% (count) and 70%–100% (weighted count) of any phase of Kote-bu, Men-bu and Do-bu, and Kamae of Tsuki-bu (Table 1), the left-hand pressure was greater than the right-hand pressure. The exception was the Datotsu phase of Tsuki-bu, where the left-hand pressure was greater than the right-hand pressure in only 18.50% (count) and 11.73 (weighted count).

Weighted numbers that were greater than non-weighted numbers indicated that |Δ*p*_L–R_| was greater if Δ*p*_L–R_ was positive (i.e., left-hand pressure greater than right-hand pressure), except for the Tsuki-bu thrust, where weighted numbers that were smaller than non-weighted numbers indicated that |Δ*p*_L–R_| was greater if Δ*p*_L–R_ was negative (i.e., right-hand pressure greater than left-hand pressure). 

The left-hand pressure was greater than the right-hand pressure in all five phases with the highest percentage (42.7–44.2%; Table 2), for scoring targets of Kote-bu, Men-bu, and Do-bu. This was followed by the left-hand pressure being greater than the right-hand pressure (24–39%) in four out of five phases. In the Tsuki-bu target, there were only two phases (Kamae, and Datotsu); consequently, there were only three combinations: right-hand pressure greater than left-hand pressure in both phases in 18.6%; left-hand pressure greater than right-hand pressure in both phases in 16.3%; and right-hand pressure greater than left-hand pressure in one phase in 63.3%.

The player dependency of the pressure distribution is shown in Table 3. 

Table 3 refers to Hypothesis 4 (referring to the specific player-dependent variation of the grip pressure), which is clearly different from Hypothesis 2 (referring to the averaged data). Therefore, when combining all Kendokas (23) and all five attack phases, we obtained 115 data points for each of the three attack targets of Kote, Men, and Do per hand. Let 115 be 100%, then Table 3 reports the percentage of how many of the 115 data points showed a greater left-hand pressure and a smaller right-hand pressure.

The percentage of players that exhibited a greater left-hand pressure compared to the right-hand pressure was grouped in four percentage quarters (left-hand pressure greater in 0–25%, 26–50%, 51–75%, or 76–100% of all phases). For scoring targets of Kote-bu, Men-bu, and Do-bu, there were no players in the first quarter, and the maximum number of players were in the last quarter (65–83% of all players). In the Tsuki-bu target, the maximum number of players was found in the second quarter, mainly because of opposite pressure distribution of the two phases, i.e., higher left-hand pressure in the Kamae and higher right-hand pressure in the Datotsu phase.

Hypothesis 4 was therefore confirmed in most cases, given the fact that the left-hand pressure was greater in 60.22–100% (count) and 70–100% (weighted count) of any phase of Kote-bu, Men-bu, Do-bu, and Tsuki-bu, with the exception of the Datotsu phase of Tsuki-bu; the left-hand pressure was greater in 42.7–44.2% of all five phases of Kote-bu, Men-bu, and Do-bu; and 65–83% of all players had a greater left-hand pressure in 76–100% of all phases.

The summary of all attack phases with motion and grip pressure observations is detailed in Table 4.

## 4. Discussion

Based on pressure measurement data, this study provides for the first time the proof that the hypothetical and empirical Kendo attack phases do exist. It is obvious from the grip pressure data that the left hand is the one that produces more pressure in general for the Kendo stance (Kamae) and scoring targets such as Kote-bu, Men-bu, and Do-bu. This result is aligned to the theory taught by most Kendo instructors who believe that the Kendokas should focus on the left hand rather than on the right hand [3]. This belief is in line with the results of this study, as well as with a previous one [11], but stands in sharp contrast to two other studies [8,9] who measured a greater pressure in the right hand. 

The coordination of the left and right hands is observed from the similarity in the grip pressure patterns, however, with significantly different pressure values of left and right hands in all the attack scenarios. This is expected as the coordination of the left and right hands is one of the key factors for effective cuts in Kendo. According to the survey results from Kendo Sensei(s), the effective transition of grip pressures between left and right hands from Kamae to Datotsu is important to produce effective cuts. In Kamae, the grip pressure is at the minimum as it is the basic stance. The low pressure requires less energy expenditure; hence the practitioner can maintain this stance for a longer time. This low pressure also provides the flexibility required to initiate attacks on the different targets. Therefore, the low grip pressure of the Kamae phase is observed at the beginning of all the attacking motions. 

The point-scoring motions in Kendo can be separated into three main categories, which are the striking motion in Do-bu, thrusting plus striking in Kote-bu and Men-bu, and thrusting motion (Tsuki-bu). The grip pressure patterns showed clear differences between the striking motions (Figure 5a–c) and thrusting motion (Figure 5d). In all striking motions (Kote-bu, Men-bu, and Do-bu), five phases of grip pressures were observed. These were Kamae (basic stance), Seme (attack initiation), Toraeru (grab an opportunity), Datotsu (impact), and Zanshin (display of strong fighting spirit and readiness for defence or further attack). The grip pressure increased significantly from Kamae to the Seme as the Kendokas initiated the cut by pushing the Shinai outward to reach for the target. The drop in the grip pressure observed during the Toraeru phase was due to the Kendoka deciding on the attack opportunity. This was usually explained by Japanese Kendo experts as ‘catching an opportunity’. At this stage, the Kendoka decides to attack or withdraw. If an attack is initiated, the Kendoka has to decide which target to attack. Therefore, the grip must be loosened to allow the change in direction. The grip pressure increased again during the Datotsu. The high grip pressure was still observed in the Zanshin phase (or follow-through motion) as the Kendokas must exhibit a strong fighting spirit after an attack by continuing the attacking posture for a few more seconds. This is one of the essential scoring criteria. 

The grip pressure pattern in the Men-bu target found in this study was also similar to the grip pressure pattern reported by Watanabe et al. [9]. In addition, the observed pressure pattern of striking motions in Kendo was like the grip pressure pattern of a professional golf player reported by Broker and Ramey [22]. They presented data that confirmed the variation of the grip force and grip pressure distribution during the swing. The total grip force peaked immediately after the top of the backswing, reached a local minimum near the impact, and increased after impact. A similar pattern was also observed in the grip pressure study of the tennis forehand [23] and backhand drives [24]. 

In the thrusting motion of the Tsuki-bu target, only two stages of grip pressure, which are Kamae and Datotsu, were observed. The grip pressure in the Kamae was like the Kamae pattern in other attacking motions. This is expected as Kamae is the basic stance for every attack. The Tsuki-bu thrusting motion requires no change in hand direction as the Kendoka only thrusts the tip of the Shinai toward the throat of the opponent. As in the Kamae stance, the tip of the Shinai is already pointing toward the throat of the opponent. Therefore, the Tsuki-bu motion only requires the extension of both arms from the Kamae in the straight direction. Therefore, neither Seme nor Toraeru phases of grip pressure were observed. The higher grip pressure of the right hand during the Datotsu of the Tsuki-bu can be explained by the right hand being used as the guiding hand for applying the thrust force on the target.

From the timing of the attack phases (Figure 4), we concluded that the varying grip pressure is controlled implicitly by the participants, as the three core attack phases, Seme, Toraeru, and Datotsu, are executed within less than 0.5 s when attacking the Kote-bu, Men-bu, and Do-bu targets. The pressure of the Toraeru phase was consistently and significantly different from the pressure of the Seme or Datotsu phases across both hands in the Kote-bu, Men-bu, and Do-bu targets across all participants. As the teaching of the five attack phases was uncommon in Kendo, in combination with the results that the variation of the grip pressure was carried out in a consistently similar way across all participants, we deduced that the grip pressure variation was the result of a natural motion pattern.

The limitations of our study were twofold:

(1) The data sampling frequency of 50 Hz seemed to be very low for this kind of study. Nevertheless, the minimum number of data points we obtained in any phase was three, which was sufficient for identifying anti-persistent events (peaks, troughs) or persistent ones such as trends, and therefore suitable for detecting the different phases.
(2) The Kendo experience of the participants seems to be widespread. In Kendo, the Dan grades, equivalent to the black belt in other martial arts, are considered “expert/elite” in the Kendo field. Therefore, any practitioner with 1st Dan and above has the necessary and sufficient skill levels as any higher Dan grade has. Moreover, we detected the same data pattern shown in Figure 5 in both lower and higher Dan grade holders.

Based on the statistical trends we obtained from our results, we recommend that Sensei teach the following grip pressure guidelines when coaching a Kendo class:

(1) In the basic stance (Kamae) before launching an attack, the left hand exerts a greater pressure to the grip of the Shinai than the right hand does.
(2) When attacking the targets of Kote-bu, Men-bu, and Do-bu, the left hand exerts a greater pressure.
(3) When attacking the Tsuki-bu, the right hand exerts a greater pressure.
(4) While there is only one attack phase in the Tsuki-bu (namely the Datotsu), there are four attack phases in the other three attack targets: Seme, Toraeru, Datotsu, and Zanshin.
(5) In the Seme, the left-hand pressure is the greatest, in the Toraeru the lowest, and in Datotsu and Zanshin, the pressure is at a medium level.

The Kendokas should practise the pressure variations from Kamae to Seme, Toraeru, Datotsu, and Zanshin, and back to Kamae, in slow motion.

## 5. Conclusions

Different grip pressure patterns were observed for striking motion (Kote-bu, Men-bu, and Do-bu) and thrusting motion (Tsuki-bu). A firm left hand grip is required in most Kendo motions. The right hand was observed to be used for direction control and the right-hand grip pressure was also the essential requirement for decelerating the Shinai after successful cuts (Tenouchi). In striking motions, the grip pressure can be divided into five stages. This pattern is comparable to the grip pressure pattern reported for tennis and golf swings. In the thrusting motion, due to the unchanged direction of the moving hands, only two grip pressure stages were observed. 

## Figures and Tables

**Table 1:** Percentage of instances with left-hand pressure greater than right-hand pressure across all phases per attack technique of 10 attacks per technique and participant; relative numbers (%) and relative weighted numbers (%, higher pressure has greater weight).

	Kote-bu (Numbers) %	Kote-bu (Weighted Numbers) %	Men-bu (Numbers) %	Men-bu (Weighted Numbers) %	Do-bu (Numbers) %	Do-bu (Weighted Numbers) %	Tsuki-bu (Numbers) %	Tsuki-bu (Weighted Numbers) %
Kamae	78.47	86.29	85.96	90.43	79.89	91.26	78.85	91.32
Seme	96.36	99.54	100	100	88.34	97.08	--	--
Toraeru	90.14	97.07	84.364	96.35	92.50	97.39	--	--
Datotsu	62.00	71.76	83.80	94.86	60.22	70.00	18.50	11.73
Zanshin	85.97	94.03	68.72	82.31	82.22	86.90	--	--

**Table 2:** Percentages of combinations of left and right grip pressures; *p* = pressure.

Attack Technique	Kote-bu (Percentage of Phase Combination)	Men-bu (Percentage of Phase Combination)	Do-bu (Percentage of Phase Combination)
all 5 phases: *p*_R_ > *p*_L_	0.5	0	1.2
4 phases: *p*_R_ > *p*_L_; 1 phase *p*_L_ > *p*_R_	4.1	0	2.3
3 phases: *p*_R_ > *p*_L_; 2 phases *p*_L_ > *p*_R_	3.6	5.2	4.7
3 phases: *p*_L_ > *p*_R_; 2 phases *p*_R_ > *p*_L_	7.7	8.1	18.7
4 phases: *p*_L_ > *p*_R_; 1 phase *p*_R_ > *p*_L_	31.1	39.0	24.0
all 5 phases: *p*_L_ > *p*_R_	44.1	44.2	42.7
remaining combinations	8.9	3.5	6.4

**Table 3:** Player-dependent percentages of pressure distribution across attack phases (five in Kote-bu, Men-bu, and Do-bu; two in Tsuki-bu).

Percentage of Players That Exhibited Greater Pressure in the Left Hand Compared to the Right Hand in…:	Kote-bu (Percentage of Players)	Men-bu (Percentage of Players)	Do-bu (Percentage of Players)	Tsuki-bu (Percentage of Players)
… 0–25% of all phases	0	0	0	13
… 26–50% of all phases	13	0	9	61
… 51–75% of all phases	9	17	26	17
… 76–100% of all phases	78	83	65	9

**Table 4:** Summary of the five attack phases in terms of motions and associated grip pressure.

Phase (Japanese)	Phase (English Explanation)	Associated Motion Explained for Coaching Purposes	Grip Pressure Observation
Kamae (構える)	Basic stance or normal stance.	Left hand positioned at the end of the Tsukagawa (grip handle butt) with right hand closer to the hand guard (Tsuba).	Low stable grip pressure of both hands with slightly higher grip pressure in the left hand.
Seme (攻める)	Attack initiation phase. Seme means ‘to attack’, but in Kendo it refers to breaking through the opponent’s defence.	The Kendoka applies pressure to the opponent usually through forcing the Shinai forward to try to break the opponent’s centre.	The grip pressure increases and shows a spike coincidental with the initial acceleration of the Shinai.
Toraeru (捉える)	Phase for catching an opportunity. After the Kendoka goes through the Seme phase, he/she observes the reaction of the opponent before deciding on the attack.	The Kendoka observes the opponent’s reaction. If the opponent does not lower the defence, then the Kendoka usually withdraws and starts the process of Seme again. If the offence with Seme is successful, the attack is launched.	The pressure drops, temporarily and significantly, before the strike phase.
Datotsu (打突する)	Strike phase Correct part of the Shinai hits the target.	The moment the correct part of the Shinai hits the target. This is when the Tenouchi (use of wrist and hand grip to manoeuvre the Shinai effectively) is emphasised. This usually refers to the ‘wrist snapping’ motion during the impact.	The pressure increases again and shows a spike; the spike is a combination of increasing grip pressure as well as of the impact spike acting on the Shinai and therefore acts also on both hands.
Zanshin (残心を示す)	Follow Through Display of strong fighting spirit and maintaining basic stance to be ready for defence or further attack.	This is the motion after the strike. The Kendoka exhibits a strong spirit by passing through the opponent with a completed strike motion and returns to the Kamae at a safe distance.	The pressure between the impact and follow through decreases slightly, but in general, the pressure remains high and directly continues into the follow-through pressure hump.

## Data Availability

The data presented in this study are available on request from the corresponding author to any qualified researcher, if they have obtained Ethics Approval for secondary use of existing data through a Consent Waiver.
